# Triglyceride-glucose index variability and incident cardiovascular disease: a prospective cohort study

**DOI:** 10.1186/s12933-022-01541-5

**Published:** 2022-06-10

**Authors:** Haibin Li, Yingting Zuo, Frank Qian, Shuohua Chen, Xue Tian, Penglian Wang, Xia Li, Xiuhua Guo, Shouling Wu, Anxin Wang

**Affiliations:** 1 Department of Cardiac Surgery, Heart Center & Beijing Key Laboratory of Hypertension, Beijing Chaoyang Hospital, Capital Medical University, Beijing, China; 2 Beijing Advanced Innovation Center for Big Data-Based Precision Medicine, Capital Medical University, Beijing, China; 3 Department of Neurology, Beijing Tiantan Hospital, Capital Medical University, Beijing, China; 4 China National Clinical Research Center for Neurological Diseases, Beijing Tiantan Hospital, Capital Medical University, Beijing, China; 5 Department of Epidemiology and Health Statistics, School of Public Health, Capital Medical University, Beijing, China; 6 Department of Medicine, Beth Israel Deaconess Medical Center, Harvard Medical School, Boston, MA USA; 7 Department of Cardiology, Kailuan Hospital, North China University of Science and Technology, Tangshan, China; 8 Department of Mathematics and Statistics, La Trobe University, Melbourne, VIC Australia

**Keywords:** Triglyceride-glucose index, Cardiovascular disease, Variability, Cohort study

## Abstract

**Background:**

Recent studies have suggested that triglyceride-glucose (TyG) index is an independent predictor of cardiovascular disease (CVD). However, the impact of long-term visit-to-visit variability in TyG index on the risk of CVD is not known. We aimed to investigate the longitudinal association between baseline and mean TyG index as well as TyG index variability and incident CVD in a Chinese population.

**Methods:**

We included 49,579 participants without previous history of CVD in the Kailuan study who underwent three health examinations (2006, 2008, and 2010) and were followed up for clinical events until 2019. TyG index was calculated as Ln [fasting triglyceride (mg/dL) × fasting glucose (mg/dL)/2]. We measured TyG index variability as the SD of the residuals obtained from a linear regression on the three TyG index measurements for each individual. Multivariate-adjusted Cox models were used to estimate the adjusted hazard ratio (aHR) and 95% confidence interval (CI) with incident CVD.

**Results:**

During a median follow-up time of 9.0 years, 2404 developed CVD. The highest tertile (T3) of baseline and mean TyG index were each associated with higher CVD incidence as compared with the lowest tertile (T1): aHR, 1.25; 95% CI 1.11–1.42; and aHR 1.40; 95% CI 1.24–1.58, respectively. Tertile 3 of TyG index variability was associated with increased CVD incidence compared to T1 group (aHR, 1.12; 95% CI 1.01–1.24). Similar findings were observed in a series of sensitivity analyses.

**Conclusion:**

Higher TyG index level and greater TyGindex variability were each independently associated with a higher incidence of CVD.

**Supplementary Information:**

The online version contains supplementary material available at 10.1186/s12933-022-01541-5.

## Background

Insulin resistance has been identified as an important risk factor for the development of cardiovascular disease (CVD) [1], which is a leading cause of morbidity and mortality in China and worldwide [2, 3]. A meta-analysis of cohort studies or nested case–control studies have shown a positive prospective relationship between insulin resistance and risk of CVD in non-diabetic adults [4]. Furthermore, a Mendelian randomization analysis suggested a causal relationship between the insulin resistance and CVD [5]. Therefore, the early identification of individuals with insulin resistance  will be essential to reduce the disease burden of CVD.

In the clinical setting, measurement of insulin resistance can be challenging as there are limitations to the homeostasis model assessment for insulin resistance (HOMA-IR) and the gold standard of the euglycemic clamp is time-consuming and burdensome [6]. The triglyceride-glucose (TyG) index, which is the logarithmized product of fasting triglyceride and glucose, has been shown to be a simple measure of insulin resistance [7]. Previous studies have shown that TyG index is significantly related to an increased risk of cardiovascular events [8–16]. Additionally, a recent meta-analysis of cohort studies that included 5,731,294 participants without CVD at baseline showed that highest TyG index category was associated with 1.61-fold increased risk of CVD [17]. However, most of prior studies were based on a single baseline TyG index measurement [8–12], which may not reflect long-term exposure. Few studies have examined repeated TyG measurements to evaluate the impact of longitudinal TyG index on the risk of CVD. To our knowledge, only one study examined the association between change in TyG index at two time points and incident CVD [14]. Additionally, three studies have reported on the association between time updated average TyG index and the number of visits with a high TyG index and CVD events [8, 9, 13]. Furthermore, although TyG index may change over time, to our knowledge, no study has examined the association between long-term TyG index visit-to-visit variability and CVD development.

Visit-to-visit variability in cardiovascular risk factors has been shown to predict the risk of adverse long-term cardiovascular outcomes and mortality, independent of baseline or average risk factors levels over [18]. Recently, the association between visit-to-visit variability in various biological measures (e.g., blood pressure, cholesterol, and glucose) and incident CVD have been examined [19–21]. Our previous study has also shown that high long-term glycemic variability, measured as intraindividual variability in fasting glucose over time was associated with cardiovascular outcomes, independently of mean glucose levels [22]. Additionally, some studies found associations between greater variability in lipid measurements with cardiovascular outcomes [19–21, 23, 24]. Although TyG index is derived from fasting triglyceride and glucose, variability in TyG index is not uncommon. Whether visit-to-visit variability in TyG levels affect future cardiovascular outcomes in the general population is not known. Moreover, such studies can provide further evidence for future interventional trials that focus on reducing insulin resistance variability in addition to average insulin resistance levels.

Therefore, we conducted a large population-based study involving more than 49,000 Chinese adults who had repeated measurements in TyG index to investigate the longitudinal association between baseline TyG level, visit-to-visit variability in TyG index, and CVD incidence during a median 9.0-year follow-up in a general population. We hypothesized that higher TyG level as well as greater variability are both associated with increased risk of CVD.

## Study population

Details of the Kailuan study cohort design, methods, and data collection have been published previously [8, 9, 14, 19]. In brief, the Kailuan study recruited 101,510 community-dwelling adults aged 18 years and over between June 2006 and October 2007 in the Kailuan community, Tangshan City, China. A standardized interview and health examinations were conducted at baseline and follow-up. This study was approved by the Ethics Committee of Kailuan General Hospital and Beijing Tiantan Hospital, Capital Medical University, and it was conducted according to the principles of the Declaration of Helsinki. All participants provided written informed consent.

**Fig. 1:**
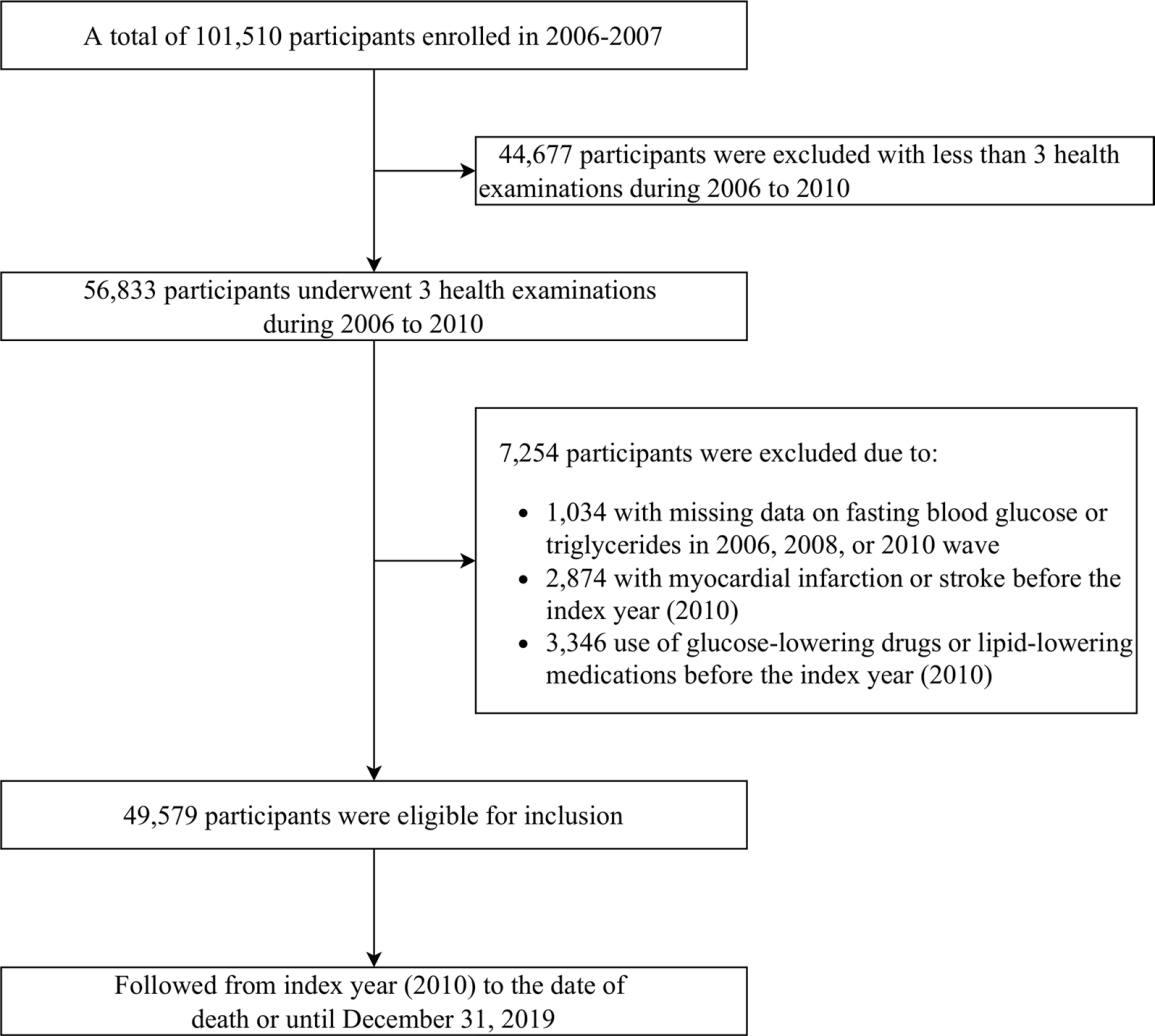
Flowchart of participants selection

In this analysis, we included participants who underwent 3 health examinations between June 2006 and December 2010 (baseline and index year). Of 56,833 participants, we excluded those who had missing data on fasting blood glucose or triglycerides (n = 1034), those who had a previous diagnosis of stroke or myocardial infarction (n = 2874), and those who were on either lipid-lowering or antidiabetic drugs (n = 3346) during the 4-year exposure assessment period. Therefore, 49,579 eligible participants were included in the current study (Fig. 1**)**. The study design was displayed in the Additional file 1: Fig. S1.

### Data collection and definitions

Information on demographics, lifestyles, medical histories, anthropometric measurements, and laboratory tests were collected at baseline and follow-up surveys, as detailed elsewhere [8, 9, 14, 19]. In brief, information on age, sex, education level, average monthly income, smoking status, alcohol intake, physical activity, and past self-reported medical history (e.g., hypertension and diabetes) and medication use (e.g., hypoglycemic agents, antihypertensive agents, and lipid-lowering agents) was collected via a face-to-face validated questionnaire. Education level was categorized into two groups: low (illiteracy or primary school, or middle school), and high (high school or college/university). The low-income level was defined as participants’ average monthly income ≤ 1000 yuan. Participants who currently smoked were defined as current smokers, and those who currently drank were defined as current drinkers. Physical activity was assessed by questionnaire according to lifestyle behaviors that combined occupational and discretionary physical activities and divided into “physically inactive” and “physically active”. Trained physicians or nurses measured participants’ height, weight, systolic blood pressure (SBP), and diastolic blood pressure (DBP). Body mass index (BMI) was calculated as weight (kg)/height (m)^2^. Fasting blood samples were collected and were measured using the Hitachi 747 auto-analyzer (Hitachi, Tokyo, Japan). Serum total cholesterol (TC), triglycerides (TG), high-density lipoprotein cholesterol (HDL-C), low-density lipoprotein cholesterol (LDL-C), fasting blood glucose (FBG), creatinine, and high-sensitivity C-reactive protein (hs-CRP) were measured via a standardized protocol. Hypertension was defined as a self-reported history of hypertension, use of antihypertensive medications, SBP ≥ 140 mmHg, or DBP ≥ 90 mmHg. Diabetes was defined as self-reported history of diabetes or FBG ≥ 7.0 mmol/L. Hypercholesterolemia was defined as self-reported history of dyslipidemia, or TC ≥ 5.17 mmol/L. Estimated glomerular filtration rate (eGFR) was calculated using the Chronic Kidney Disease Epidemiology Collaboration creatinine equation [25]. Chronic kidney disease was defined as an eGFR < 60 mL/min/1.73 m^2^. High hs-CRP was defined as a hs-CRP > 3 mg/dL [26].

### Definition of baseline, mean and variability of TyG index

According to previous studies [8, 14], the TyG index was calculated as Ln[TG (mg/dL) × FBG (mg/dL)/2]. The baseline TyG index was calculated by using serum TG and FBG measured in 2010. Mean TyG index was defined as the average of TyG index measured in 2006, 2008, and 2010. The TyG index variability was defined as intraindividual variability in TyG levels measured on these three health examinations. As previously described [27, 28], we used residual SD, defined as visit-to-visit TyG index variability calculated as the root-mean-square error (RMSE) of the residuals (i.e., differences between observed TyG and predicted TyG) obtained from a simple linear regression analysis of the three TyG measurements of each participant.

### Study outcome and follow-up

The study outcome was newly diagnosed CVD, which was defined as a composite of myocardial infarction and stroke. As previously described [14, 29], all participants were linked to the Municipal Social Insurance Institution and the Hospital Discharge Register for incidence of CVD, which cover all the Kailuan study participants. To further identify potential CVD cases, we reviewed the discharge lists from the 11 hospitals during 2006–2019 and asked for a history of CVD via a questionnaire during the biennial interview. For all suspected CVD events, three experienced physician adjudicators who were blinded to the study design reviewed the medical records. Myocardial infarction was defined as the recording of ICD-10 codes I21. Stroke was defined as the recording of ICD-10 codes I63, or I60 to I61. The vital status was obtained from Hebei Provincial Vital Statistics Offices or directly contacting the participants’ family members and reviewed by physicians.

### Statistical analysis

Baseline characteristics of Kailuan study participants were described across TyG index variability tertiles. Continuous variables were summarized as mean ± SD or median (interquartile range) depending on variable distribution, and categorical variables as count (proportion). Continuous variables were compared using one-way ANOVA or the Kruskal–Wallis test, and categorical variables were compared using the χ^2^ test across tertiles of TyG index variability. Covariate balances were also examined using standardized mean differences (SMDs). We considered a SMDs > 0.1 as indicative of imbalance.

We analyzed risk of CVD according to the tertiles of baseline, mean, and variability of TyG index, and the combination of baseline TyG index tertiles and TyG index variability tertiles. Person-years of follow-up for each participant were calculated as the amount of time from the index date to the first of the following events: incident CVD, death, or December 31st, 2019. Incidence rate of CVD per 1000 person-years was calculated. Time to first CVD event was examined using Kaplan–Meier survival curves and compared using log-rank test. Multivariable-adjusted Cox proportional hazards regression models were used to estimate the adjusted hazard ratios (aHR) and 95% confidence intervals (CI). The proportional hazards assumption was verified by inspecting the negative log–log survival plots and no violation was observed. Model 1 was adjusted for age and sex. Model 2 was adjusted for age, sex, education, income, current smoking status, current drinking status, physical activity, BMI, diabetes, hypertension, chronic kidney disease, and hs-CRP. Model 3 was additionally adjusted for hypercholesterolemia, HDL-C, and LDL-C. Model 4 was additionally adjusted for baseline TyG index for  analyses of  TyG index variability. In the trend test, the categorical variable (i.e., TyG index variability tertile) was statistically examined as an ordinal variable (continuous variable) in Cox regression model.

The linearity of baseline TyG index, mean TyG index, and TyG index variability for CVD risk was assessed using a restricted cubic spline Cox model. The association between TyG index variability and risk of CVD were further examined when stratified by the baseline level of TyG index. Interactions between  baseline TgG index and TyG index variabllity in relation to CVD risk were assessed by likelihood ratio test. In addition, optimal cut-off points for baseline TyG index, mean TyG index, and TyG index variability associated with incident CVD were determined using an outcome-oriented method to maximize log-rank statistics [30]. Harrell’s C statistics and net reclassification improvement (NRI) for survival data were used to estimate the improvement in discrimination and reclassification after adding the baseline and TyG index variability to the clinical model [31]. 95% CI for continuous NRI were estimated with 500 bootstrap replications.

To estimate the population impact of TyG index measures on CVD risk, we estimated the absolute risk difference between baseline, mean, and variability of TyG index and incident CVD. Predicted cumulative incidence and absolute risk differences were presented as per 1000 population over 10 years and were estimated using flexible parametric survival models on the cumulative hazard scale [32], similar to what has been done previously [33]. We also plotted the adjusted cumulative incidence curves for CVD by extrapolating to 10 years using *stpm2* and *standsurv* command in Stata, which were standardized to the baseline covariates.

Several sensitivity analyses were conducted as follows: (1) we also categorized TyG index variability into five groups: < 0.15, 0.15 to < 0.30, 0.30 to < 0.45, 0.45 to < 0.60, and ≥ 0.6, which was based on visual inspection of histograms showing the population distributions of TyG index variability (Additional file 1: Fig. S2); (2) to better capture the long-term direction of variation in TyG index, we also calculated the annual increase rate for TyG index over time [the slope of the simple linear regression model in which TyG index in 2006, 2008, and 2010 was the response variable and follow-up duration (years) was the independent variable]. We categorized the slope into decreasing (< −0.2/year), stable (−0.2 to 0.2/year), and increasing (> 0.2/year) groups based on visual inspection of histograms (Additional file 1: Fig. S3); (3) we excluded participants with FBG ≥ 7.0 mmol/L or TG ≥ 1.7 mmol/L at baseline; (4) Fine-Gray competing risk regression was used, which treated deaths as competing risk events [34]; (5) 2-y lag analysis was conducted, which excluded CVD events that occurred within 2 years of follow-up; (6) other variability measures including SD, coefficient of variation (CV), and independent of the mean (VIM) were calculated [19]; and (7) to assess the influence of unmeasured confounding, E-value, which is defined as the minimum strength of association, was calculated based on the estimated HR and 95% CI for CVD [35].

Statistical analyses were conducted using STATA MP, version 16.0 (StataCorp) and R software, version 4.1.3. All *P*-values were 2-sided and a *P* < 0.05 was considered statistically significant, unless otherwise stated.

## Results

Among the 49,579 participants, the mean age was 52.5 ± 11.8 years at baseline and 37,977 (76.6%) were male. The mean baseline TyG index level of the total study population was 8.7 ± 0.7, and the median TyG index variability was 0.2 (interquartile range 0.1–0.4). Table 1 shows the baseline characteristics of the study participants according to tertiles of TyG index variability. Participants in the highest tertile of TyG index variability were slightly younger, more frequently male, current smokers and alcohol drinker, and had lower rates of regular exercise, and lower education and income. They also had higher prevalence of hypertension, diabetes, and hypercholesterolemia, and had higher mean BMI, SBP, DBP, FBG, and TG.

**Table 1:** Baseline characteristics of study participants according to the TyG variability tertiles

	Total (n = 49,579)	TyG index variability			*P* value	SMD
		Tertile 1 (n = 16,527)	Tertile 2 (n = 16,526)	Tertile 3 (n = 16,526)		
Age, years	52.5 ± 11.8	53.3 ± 12.1	52.7 ± 11.8	51.6 ± 11.5	< 0.001	0.11
Age ≥ 60 years	12,093 (24.4)	4451 (26.9)	4135 (25.0)	3507 (21.2)	< 0.001	0.11
Male sex	37,977 (76.6)	12,413 (75.1)	12,579 (76.1)	12,985 (78.6)	< 0.001	0.07
Education (≥ high school)	13,766 (27.9)	4633 (28.1)	4671 (28.4)	4462 (27.1)	0.028	0.03
Income (≤ 1000 RMB/month)	22,510 (46.5)	7323 (45.3)	7521 (46.6)	7666 (47.7)	< 0.001	0.03
Current smoker	16,901 (34.2)	5397 (32.8)	5552 (33.7)	5952 (36.2)	< 0.001	0.06
Alcohol drinker	17,589 (35.6)	5626 (34.2)	5704 (34.6)	6259 (38.0)	< 0.001	0.08
Physical activity	6857 (13.9)	2448 (14.9)	2251 (13.7)	2158 (13.1)	< 0.001	0.03
Hypertension	22,298 (45.0)	7403 (44.8)	7333 (44.4)	7562 (45.8)	0.030	0.02
Diabetes	3154 (6.4)	919 (5.6)	932 (5.6)	1303 (7.9)	< 0.001	0.09
Hypercholesterolaemia	19,289 (38.9)	6435 (38.9)	6326 (38.3)	6528 (39.5)	0.074	0.02
Chronic kidney disease	3040 (6.1)	1075 (6.5)	1027 (6.2)	938 (5.7)	0.006	0.03
Antihypertensive agents	4548 (9.3)	1591 (9.7)	1509 (9.2)	1448 (8.9)	0.026	0.02
Body mass index, kg/m^2^	25.0 ± 3.4	25.0 ± 3.4	25.0 ± 3.4	25.1 ± 3.3	0.001	0.04
Body mass index ≥ 25 kg/m^2^	24,984 (50.4)	8276 (50.1)	8246 (49.9)	8462 (51.2)	0.036	0.02
Systolic blood pressure, mm Hg	130.1 ± 19.0	130.1 ± 19.3	129.9 ± 18.9	130.3 ± 18.7	0.113	0.02
Diastolic blood pressure, mm Hg	84.2 ± 10.8	83.9 ± 10.8	84.0 ± 10.8	84.6 ± 10.7	< 0.001	0.06
Fasting glucose, mmol/L	5.5 ± 1.5	5.4 ± 1.2	5.4 ± 1.1	5.6 ± 2.0	< 0.001	0.09
Estimated glomerular filtration rate	90.7 ± 19.9	90.0 ± 19.7	90.8 ± 20.2	91.2 ± 19.9	< 0.001	0.04
Total cholesterol, mmol/L	5.0 ± 1.3	5.0 ± 1.2	5.0 ± 1.1	5.0 ± 1.4	0.108	0.02
LDL-C, mmol/L	2.6 ± 0.9	2.6 ± 0.9	2.6 ± 0.8	2.6 ± 1.2	0.021	0.02
HDL-C, mmol/L	1.6 ± 0.5	1.6 ± 0.5	1.6 ± 0.6	1.5 ± 0.5	< 0.001	0.07
Triglycerides, mmol/L	1.3 (0.9, 1.9)	1.2 (0.9, 1.8)	1.2 (0.9, 1.8)	1.4 (0.9, 2.2)	< 0.001	0.21
Hs-CRP, mg/dL	1.0 (0.5, 2.4)	1.0 (0.5, 2.4)	1.0 (0.5, 2.4)	1.0 (0.5, 2.5)	0.391	0.01
Hs-CRP > 3 mg/dL	9662 (19.7)	3216 (19.7)	3162 (19.3)	3284 (20.2)	0.143	0.02
Baseline TyG index	8.7 ± 0.7	8.6 ± 0.6	8.6 ± 0.6	8.8 ± 0.7	< 0.001	0.22
Mean TyG index	8.6 ± 0.6	8.6 ± 0.5	8.6 ± 0.5	8.7 ± 0.6	< 0.001	0.27
Variability of TyG index	0.2 (0.1, 0.4)	0.1 (0.0, 0.1)	0.2 (0.2, 0.3)	0.5 (0.4, 0.7)	< 0.001	2.24

Values are presented as number (%), mean ± SD, or median (interquartile range)

*TyG index* triglyceride-glucose index, *LDL-C* low-density lipoprotein cholesterol, *HDL-C* high-density lipoprotein cholesterol, *Hs-CRP* high-sensitivity C-reactive protein, *SMD* standardized mean difference

### Association of baseline, mean TyG index, and TyG index variability with incident CVD

During a median 9.0 years of follow-up (425,859.9 person-years) after the TyG index variability assessment period, 2404 (4.9%) participants developed CVD. The overall incidence rate of CVD was 5.6 per 1000 person-years.

Table 2 presents the association between baseline TyG index, mean TyG index, and TyG index variability with risk of CVD. In Model 3, which was adjusted for all covariates, the baseline and mean TyG index was positively associated with the risk of CVD. The aHR of CVD in the highest baseline TyG tertile was 1.25 (95% CI 1.11–1.42) compared to that in the lowest tertile of baseline TyG index (*P* for trend < 0.001). The CVD risk of highest tertile of mean TyG index level was higher (aHR, 1.40; 95% CI 1.24–1.58) than that of lowest tertile of mean TyG level (*P* for trend < 0.001). After adjusting for all covariates, including baseline TyG index (Model 4), participants in the highest tertile of TyG index variability showed an increased risk of CVD (aHR, 1.12; 95% CI 1.01–1.24) compared to those in the lowest tertile (*P* for trend = 0.03). We observed a linear relationship of baseline TyG index, mean TyG index, and TyG index variability with incident CVD using restricted cubic splines regression (Additional file 1: Fig. S4).

**Table 2:** Association between tertiles of baseline, mean, and variability of TyG index and incidence of cardiovascular disease

	Hazard ratio (95% CI)^a^			*P* for trend^b^
	Tertile 1	Tertile 2	Tertile 3	
Baseline TyG index				
No. of cases/population	594/16532	827/16521	983/16526	
Incidence rate per 1000 person-years	4.18	5.82	6.95	
Model 1^c^	1 [Reference]	1.41 (1.27–1.56)	1.81 (1.64–2.01)	< 0.001
Model 2^d^	1 [Reference]	1.29 (1.15–1.44)	1.37 (1.22–1.54)	< 0.001
Model 3^e^	1 [Reference]	1.22 (1.09–1.37)	1.25 (1.11–1.42)	< 0.001
Mean TyG index				
No. of cases/population	548/16527	817/16526	1039/16526	
Incidence rate per 1000 person-years	3.85	5.75	7.35	
Model 1^c^	1 [Reference]	1.49 (1.34–1.66)	2.00 (1.81–2.22)	< 0.001
Model 2^d^	1 [Reference]	1.33 (1.18–1.49)	1.51 (1.35–1.70)	< 0.001
Model 3^e^	1 [Reference]	1.26 (1.13–1.42)	1.40 (1.24–1.58)	< 0.001
TyG variability				
No. of cases/population	766/16527	794/16526	844/16526	
Incidence rate per 1000 person-years	5.03	5.59	5.94	
Model 1^c^	1 [Reference]	1.07 (0.97–1.18)	1.19 (1.08–1.31)	< 0.001
Model 2^d^	1 [Reference]	1.03 (0.93–1.15)	1.13 (1.02–1.25)	0.017
Model 3^e^	1 [Reference]	1.04 (0.94–1.15)	1.13 (1.02–1.25)	0.021
Model 4^f^	1 [Reference]	1.04 (0.94–1.15)	1.12 (1.01–1.24)	0.030

*TyG* triglyceride-glucose, *CI* confidence interval

^a^The tertiles cutoff were < 8.4, 8.4 to 8.9, > 8.9 for baseline TyG index, < 8.4, 8.4 to 8.8, > 8.8 for mean TyG index, and < 0.15, 0.15 to 0.35, > 0.35 for TyG index variability

^b^*P* value from linear trend test when tertiles were treated as an ordinal variable in the Cox model

^c^Adjusted for age and sex

^d^Adjusted for age, sex, education, income, current smoking, current drinking, physical activity, body mass index, diabetes, hypertension, chronic kidney disease, and high-sensitivity C-reactive protein

^e^Adjusted for covariates in model 2 plus hypercholesterolaemia, low-density lipoprotein cholesterol, and high-density lipoprotein cholesterol

^f^Adjusted for covariates in model 3 plus baseline TyG index

**Fig. 2:**
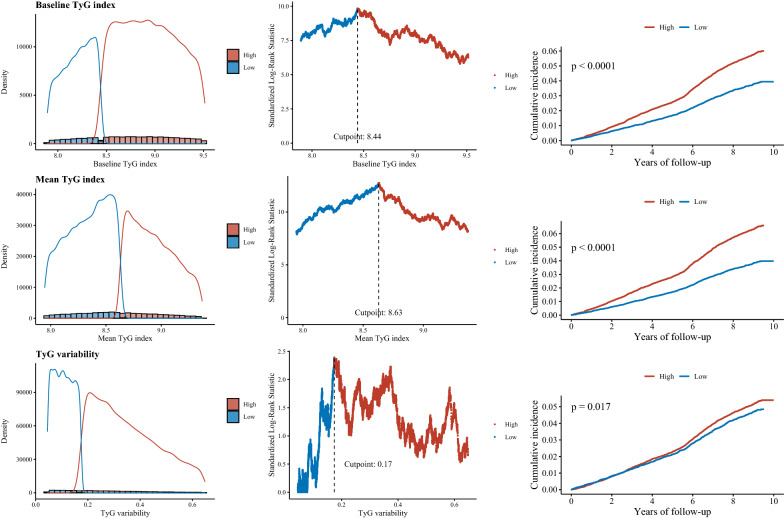
Determining cut-off values of baseline, mean, and variability of triglyceride-glucose index. Plot of the distribution (left panels), standardized log-rank statistics (middle panels), and Kaplan-Meier plot according to the cut point of baseline, mean, and variability of triglyceride-glucose index (right panels).

Using an outcome-oriented method to maximize log-rank statistics, the optimal cut-off points associated with incident CVD were ≥ 8.44 for baseline TyG index, ≥ 8.63 for mean TyG index, and ≥ 0.17 for TyG index variability (Fig. 2). The Kaplan–Meier survival curves show that the incidence of CVD risk increased with higher levels of baseline TyG index**,** mean TyG level, and TyG variability (Fig. 2).

### Associations of TyG index variability with incident CVD according to baseline TyG index level

**Fig. 3:**
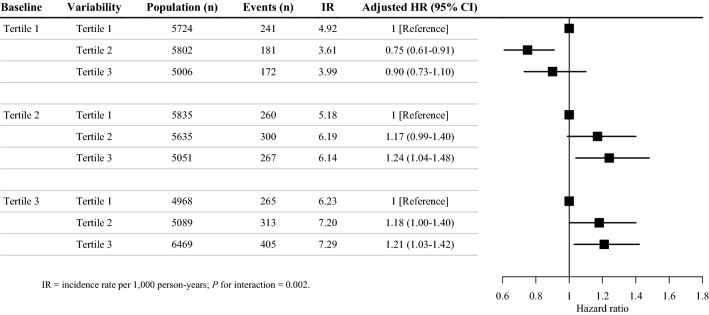
Risk of incident cardiovascular disease according to the variability and baseline level of triglyceride-glucose index

A significant interaction between TyG index variability and baseline TyG index was observed (*P* = 0.002). Figure 3 shows the associations of TyG index variability with incident CVD after stratifying participants by baseline TyG index level. Specifically, the groups with the highest incidence rate of CVD were in the highest tertile of both baseline TyG index and TyG index variability (7.29 per 1000 person-years).

### Absolute risk difference for CVD by TyG index levels and variability

**Fig. 4:**
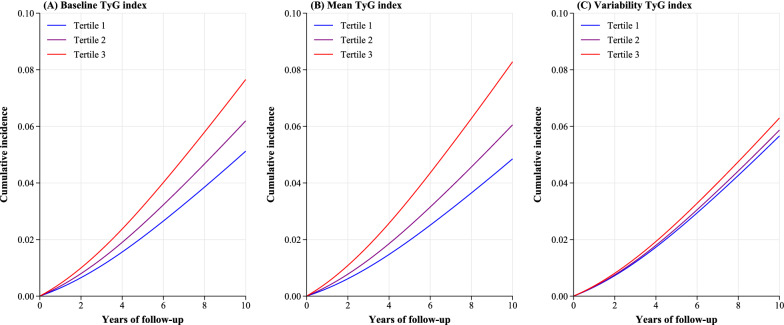
Adjusted (standardized) cumulative incidence curve for cardiovascular disease over 10-years according to baseline, mean, and variability of triglyceride-glucose index

In terms of absolute risk difference, participants in the highest tertile of baseline TyG, mean TyG, and TyG index variability were associated with 25.3 (95% CI 12.0–38.6), 34.3 (95% CI 20.2–48.3), and 6.3 (95% CI 0.6–12.1) more cases of CVD per 1000 population over 10 years, respectively, compared with participants in Tertile 1 (Table 3). The standardized cumulative incidence curves for CVD by tertiles of baseline TyG, mean TyG and TyG index variability were displayed in the Fig. 4.

**Table 3:** Absolute risk difference (per 1000 population over 10 years) of cardiovascular disease by the baseline, mean, and variability of TyG index

	Predicted incidence per 1000 population over 10 years^a^	Absolute risk difference per 1000 population over 10 years^b^
Baseline TyG index		
Tertile 1	51.2 (47.0 to 55.8)	Reference
Tertile 2	62.0 (57.9 to 66.3)	10.7 (4.8 to 16.7)
Tertile 3	76.6 (67.3 to 87.1)	25.3 (12.0 to 38.6)
Mean TyG index		
Tertile 1	48.5 (44.4 to 53.0)	Reference
Tertile 2	60.5 (56.5 to 64.9)	12.0 (6.2 to 17.8)
Tertile 3	82.8 (72.8 to 94.3)	34.3 (20.2 to 48.3)
TyG index variability		
Tertile 1	56.6 (52.8 to 60.8)	Reference
Tertile 2	58.7 (54.7 to 62.9)	2.1 (− 3.6 to 7.7)
Tertile 3	63.0 (68.9 to 67.3)	6.3 (0.6 to 12.1)

*TyG:* triglyceride-glucose

^a^Calculated as CIF_*t*_=10 × 1000, where the predicted 10-year CIF (cumulative incidence function) of CVD was estimated from the flexible parametric survival models, which was standardized to the baseline variable (covariates in Model 3 for baseline and mean TyG index, and covariates in Model 4 for TyG index variability)

^b^Calculated as the difference between the predicted incidence per 1000 population over 10 years across the baseline, mean, and variability of TyG index tertiles

### Predicting CVD risk by baseline TyG index level and TyG index variability

The addition of the baseline TyG index level and TyG index variability to a clinical model including age, sex, education, income, current smoking, current drinking, physical activity, body mass index, diabetes, hypertension, chronic kidney disease, hs-CRP, hypercholesterolaemia, LDL-C, and HDL-C increased the C-statistic from 73.56 to 73.70% (difference, 0.136%; *P* = 0.044); and there was a significant reclassification improvement (continuous NRI = 10.87%, 95% CI 6.89% to 15.27%) (Table 4).

**Table 4:** Reclassification and discrimination statistics for predicting cardiovascular disease by adding baseline and variability of TyG index

	C statistics, %	*P* value	Continuous NRI, %
	Estimate (95% CI)		Estimate (95% CI)
Clinical model^a^	73.56 (72.65–74.48)	Reference	Reference
+ Baseline TyG index	73.67 (72.76–74.58)	0.069	9.48 (5.56–13.58)
+ Baseline TyG index and variability	73.70 (72.79–74.61)	0.044	10.87 (6.89–15.27)

*TyG* triglyceride-glucose, *NRI* net reclassification improvement, *CI* confidence interval

^a^Clinical risk model included age, sex, education, income, current smoking, current drinking, physical activity, body mass index, diabetes, hypertension, chronic kidney disease, high-sensitivity C-reactive protein, hypercholesterolaemia, low-density lipoprotein cholesterol, and high-density lipoprotein cholesterol

### Sensitivity analyses

The relationship between TyG index variability and CVD remained significant when using the alternative variability categories as sensitivity analysis (aHR 1.17, 95% CI 1.02–1.34 for TyG variability > 0.6 compared to < 0.15; *P* for trend = 0.047, Additional file 1: Fig. S5). Compared to those with stable TyG index over time, both decreasing (HR: 1.13, 95% CI 0.98–1.31) and increasing groups (HR: 1.18, 95% CI 1.03–1.35) had an increased risk of CVD in the age and sex adjusted model (Additional file 1: Table S1). We did not find an association between TyG index variability when measured by SD, CV, and VIM and CVD (Additional file 1: Fig. S6). Similar association patterns were observed after excluding individuals with FBG ≥ 7.0 mmol/L or TG ≥ 1.7 mmol/L at baseline, excluding CVD events that occurred during the first 2 years of follow-up, and adjusting for competing risk of morality (Additional file 1: Table S2). E-values were 1.61, 1.84, and 1.38 for the baseline TyG index, mean TyG index, and TyG index variability (Additional file 1: Fig. S7), respectively, which suggested the associations did not influence by the potential unmeasured confounding in our study.

## Discussion

Among 49,579 Chinese adults followed up for a median of 9 years, higher baseline TyG, mean TyG, and TyG index variability were each significantly associated with a higher risk of incident CVD, and these associations persisted even after adjustment for other established cardiovascular risk factors. Overall, our data suggest that TyG index variability is an independent marker of increased CVD risk among Chinese adults.

Several prior studies have evaluated the association between baseline TyG index and cardiovascular events in the general population [8–12, 15, 16]. The Tehran Lipid and Glucose Study, with 16 years of follow-up, showed that elevated baseline TyG index was associated with a 61% and 84% increased risk of CVD and coronary heart disease, respectively [11]. In the 10-year follow-up VMCUN (Vascular Metabolic CUN) cohort, higher TyG index was associated with an increased risk of ischemic heart disease, cerebrovascular disease, and peripheral arterial disease, and TyG index could provide additional predictive value to the Framingham risk score for new-onset CVD [15], which was in accordance with our results. Another recent study with 5,593,134 persons ≥ 40 years from the Korea National Health Information Database showed that TyG index is an independent predictor of myocardial infarction and stroke during 8.2 years of mean follow-up [16]. Similar results were observed in our previous studies from the Kailuan cohort investigating the association between baseline TyG index and CVD [8, 9]. Consistent with previous studies, we confirmed that a higher TyG index at baseline was significantly associated with a 25% increased risk of future CVD.

To our knowledge, the present study is the first to investigate longitudinal associations between TyG index variability and risk of CVD in a prospective fashion. The associations we observed were independent of traditional cardiovascular risk factors as well as the baseline TyG index and thus had incremental value on CVD risk prediction. Our results indicate that participants with both high baseline and high variability in TyG index had the highest risk of CVD. This suggests the important effects of both the absolute value and the variability of TyG index in terms of the risk of CVD in the general population.

The present study demonstrated that fluctuation of TyG index level over time was also associated with incident CVD. The potential underlying mechanisms by which high TyG index variability may be associated with higher risk of CVD are still not known. We hypothesize some plausible mechanisms for why higher TyG index may associate with increased CVD risk include greater underlying metabolic dysfunction creating more dramatic changes in levels of these biomarkers (e.g., blood pressure, fasting glucose, and lipids) [20–23, 27, 36], altered lipid exchange and lipolysis , increased inflammatory response, endothelial dysfunction, and plaque instability [37–39]. Nevertheless, we considered that additional studies are needed to elucidate the mechanisms underlying the associations we observed between TyG index variability and increased risk of CVD. However, to date, there has been no consensus on a gold-standard approach to measure visit-to-visit variability. In terms of ways of assessing variability, our approach is consistent with other investigations examining visit-to-visit variability in blood pressure [27, 40], glycemia [28], and lipid levels [24] with respect to risk of incident CVD. These studies suggest that variability itself is associated with increased risk of CVD, irrespective of the particular trends over time (e.g., increasing or decreasing). In terms of clinical applications, it may be possible for contemporary electronic medical records to automatically calculate TyG index variability, though more refined methods are needed to better risk stratify individuals based on their TyG index variability over time.

Our study has several key strengths. First, the present study is the first to demonstrate an association between TyG index variability and risk of CVD. This study provides the first evidence showing that high TyG and TyG index fluctuation was independent of traditional cardiovascular risk factors. Second, this study used longitudinally and repeatedly collected TyG data at the individual level before the incidence of CVD, which allowed us to directly evaluate the long-term effect of TyG variability on CVD risk. Third, we adjusted all available confounding factors and conducted several sensitivity analyses.

This study also has some limitations. First, owing to the observational nature of the study, we could not establish a causal association between the TyG index variability and the risk of CVD. Therefore, our findings need to be confirmed in future studies. Furthermore, although potential cardiac risk factors were adjusted for, we still cannot exclude the possibility of residual or unmeasured confounding given the observational study design of the present analysis. Second, selection of study population based on the number of health examinations could be subject to selection bias. Third, most of the participants in our study were male coal miners. Hence, the findings may not be directly generalizable to the general Chinese population.

## Conclusions

In a prospective cohort of Chinese adults, we found that higher baseline TyG index, mean TyG index, as well as greater TyG variability, were each associated with higher risk of CVD. Future research is required to validate our findings and elucidate the exact mechanisms underlying the association between TyG variability and CVD.

## Supplementary Information

**Additional file 1:** **Figure S1. **Study design. ** Figure S2.** Distribution of TyG variability. **Figure S3. **Distribution of slope of TyG index. **Figure S4. **Restricted cubic spline regression for the association of baseline, mean, and variability of triglyceride-glucose index with risk of cardiovascular disease. **Figure S5. **Forest plot of adjusted hazard ratio for cardiovascular disease by TyG index variability categories. **Figure S6. **Forest plot of adjusted hazard ratio for cardiovascular disease by TyG variability according to RMSE (root-mean-square error), SD (standard deviation), CV (coefficient of variation), and VIM (independent of the mean). **Figure S7.** E-value for cardiovascular disease according to baseline, mean, and variability of TyG index. **Table S1. **Association between slope of TyG index and the incidence of cardiovascular disease. **Table S2. **Sensitivity analysis for the association between tertiles of baseline, mean, and variability of TyG index and the incidence of cardiovascular disease.

## Data Availability

The datasets used for the present analysis may be made available  upon reasonable request by contacting the corresponding author.

## Abbreviations

BMI: Body mass index
CVD: Cardiovascular disease
eGFR: Estimated glomerular filtration rate
TyG: Triglyceride-glucose
FBG: Fasting blood glucose
HDL-C: High-density lipoprotein cholesterol
LDL-C: Low-density lipoprotein cholesterol
TC: Total cholesterol
TG: Triglycerides
Hs-CRP: High-sensitivity C-reactive protein
SBP: Systolic blood pressure
DBP: Diastolic blood pressure
HR: Hazard ratio
CI: Confidence interval
