# Supplementary material for: Triglyceride-glucose index variability and incident cardiovascular disease: a prospective cohort study
Source: Cardiovasc Diabetol. 2022 Jun 10;21:105. doi: 10.1186/s12933-022-01541-5 (PMC9188105; doi:10.1186/s12933-022-01541-5)
Supplement: Supplementary file 1 — Additional file 1: Figure S1. Study design. Figure S2. Distribution of TyG variability. Figure S3. Distribution of slope of TyG index. Figure S4. Restricted cubic spline regression for the association of baseline, mean, and variability of triglyceride-glucose index with risk of cardiovascular disease. Figure S5. Forest plot of adjusted hazard ratio for cardiovascular disease by TyG index variability categories. Figure S6. Forest plot of adjusted hazard ratio for cardiovascular disease by TyG variability according to RMSE (root-mean-square error), SD (standard deviation), CV (coefficient of variation), and VIM (independent of the mean). Figure S7. E-value for cardiovascular disease according to baseline, mean, and variability of TyG index. Table S1. Association between slope of TyG index and the incidence of cardiovascular disease. Table S2. Sensitivity analysis for the association between tertiles of baseline, mean, and variability of TyG index and the incidence of cardiovascular disease. [file 12933_2022_1541_MOESM1_ESM.docx]

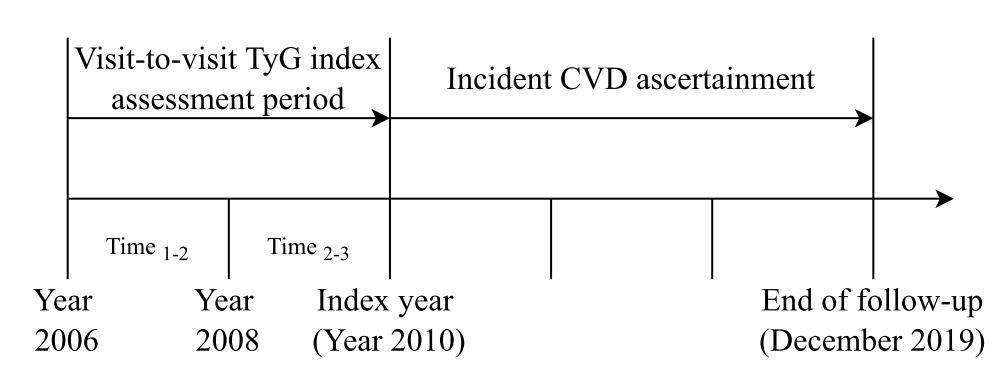


**Figure S1.** Study design. The mean time interval of time_1-2_ and time_2-3_ were 2.08 years and 1.97 years.


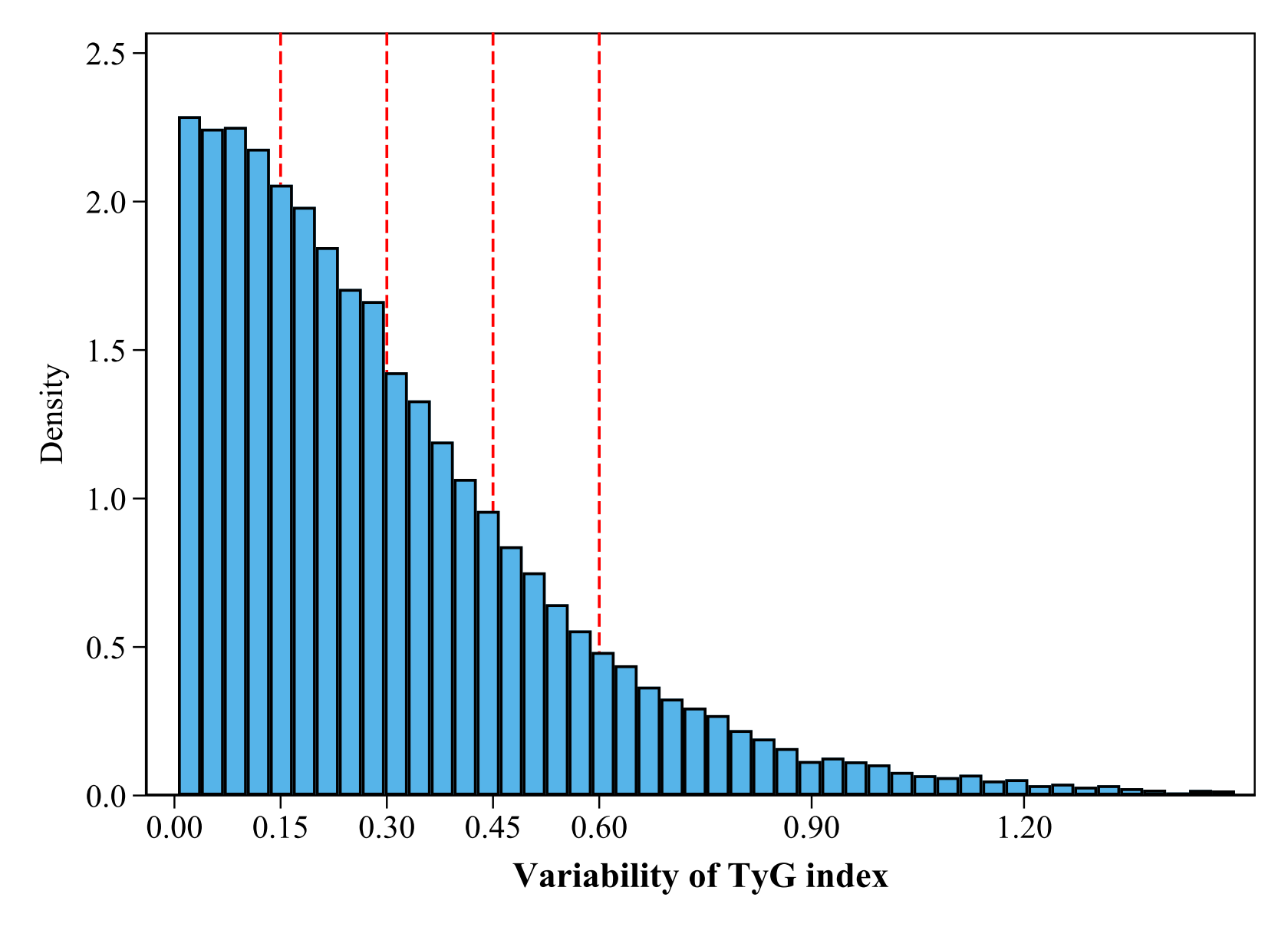


**Figure S2.** Distribution of TyG variability

**Figure S3.** Distribution of slope of TyG index

**
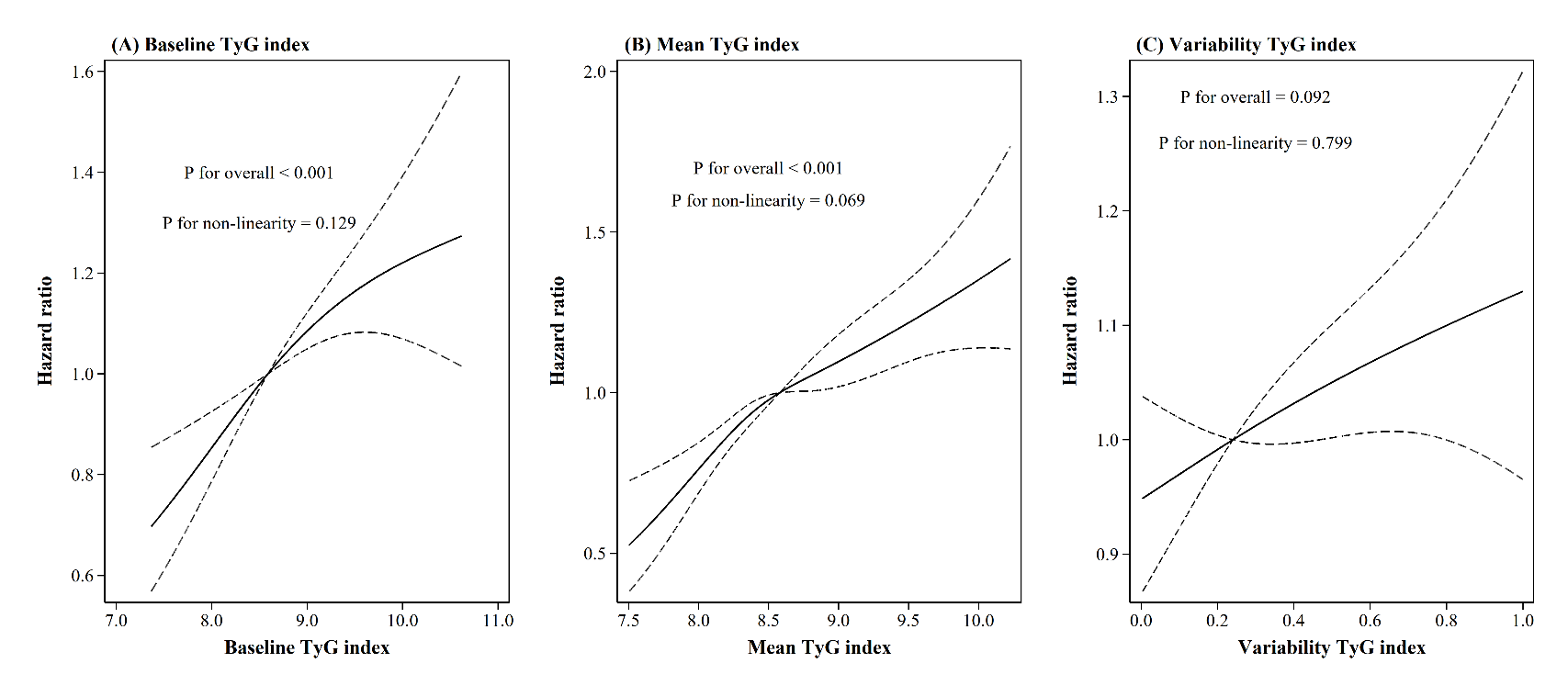
**

**Figure S4.** Restricted cubic spline regression for the association of baseline, mean, and variability of triglyceride-glucose index with risk of cardiovascular disease


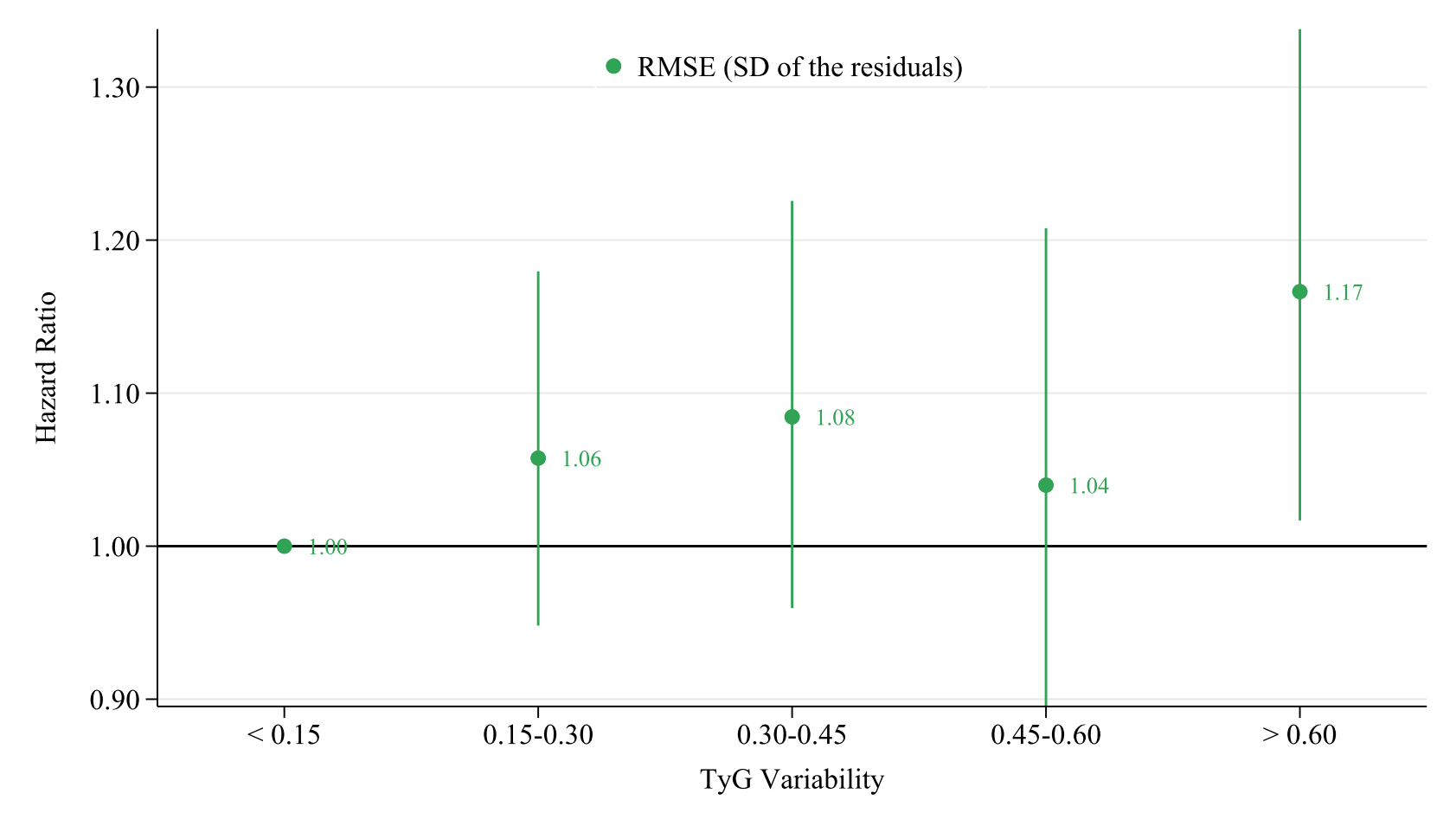


**Figure S5.** Forest plot of adjusted hazard ratio for cardiovascular disease by TyG variability categories


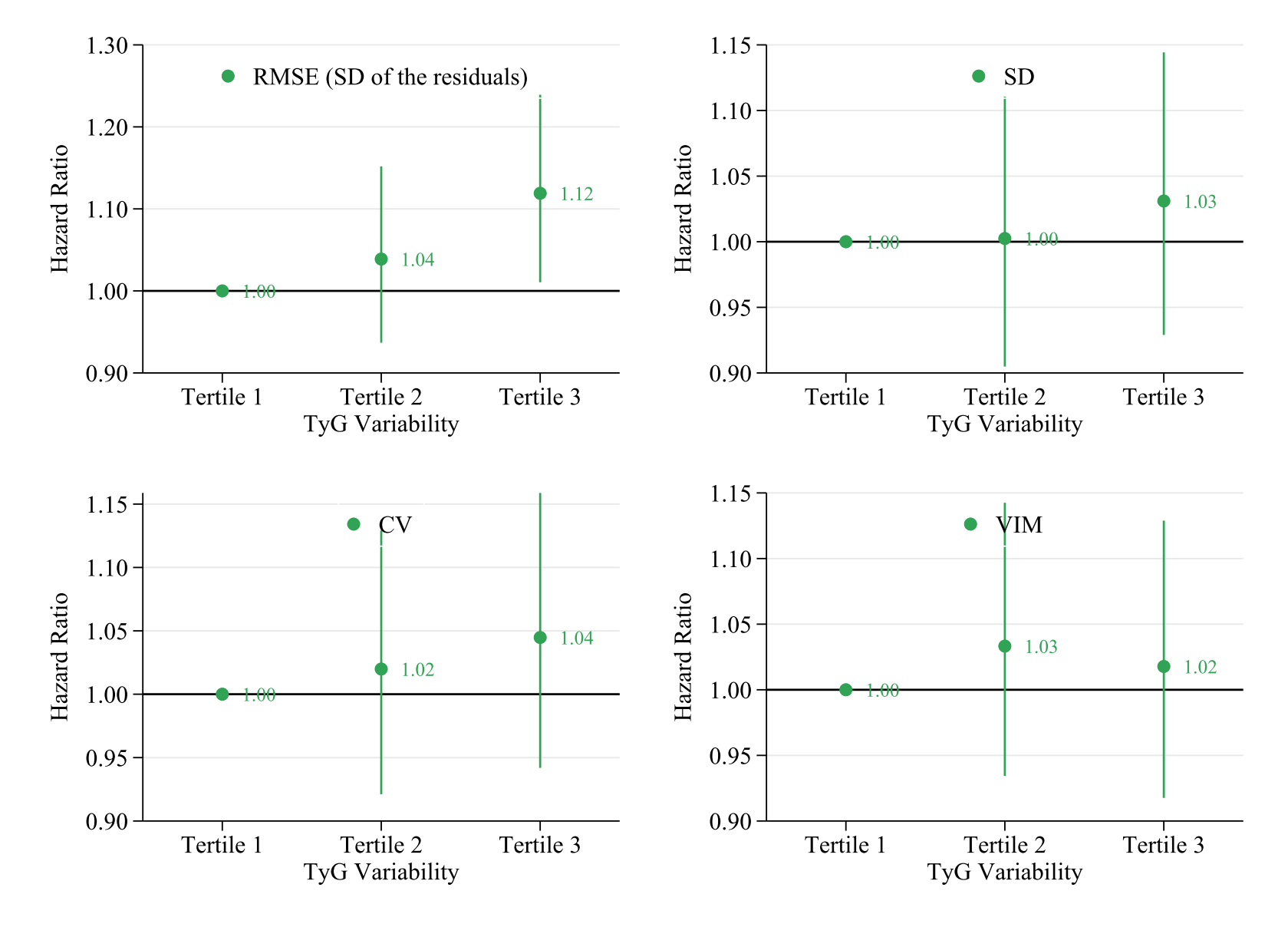


**Figure S6.** Forest plot of adjusted hazard ratio for cardiovascular disease by TgG variability according to RMSE (root-mean-square error), SD (standard deviation), CV (coeﬃcient of variation), and VIM (independent of the mean)


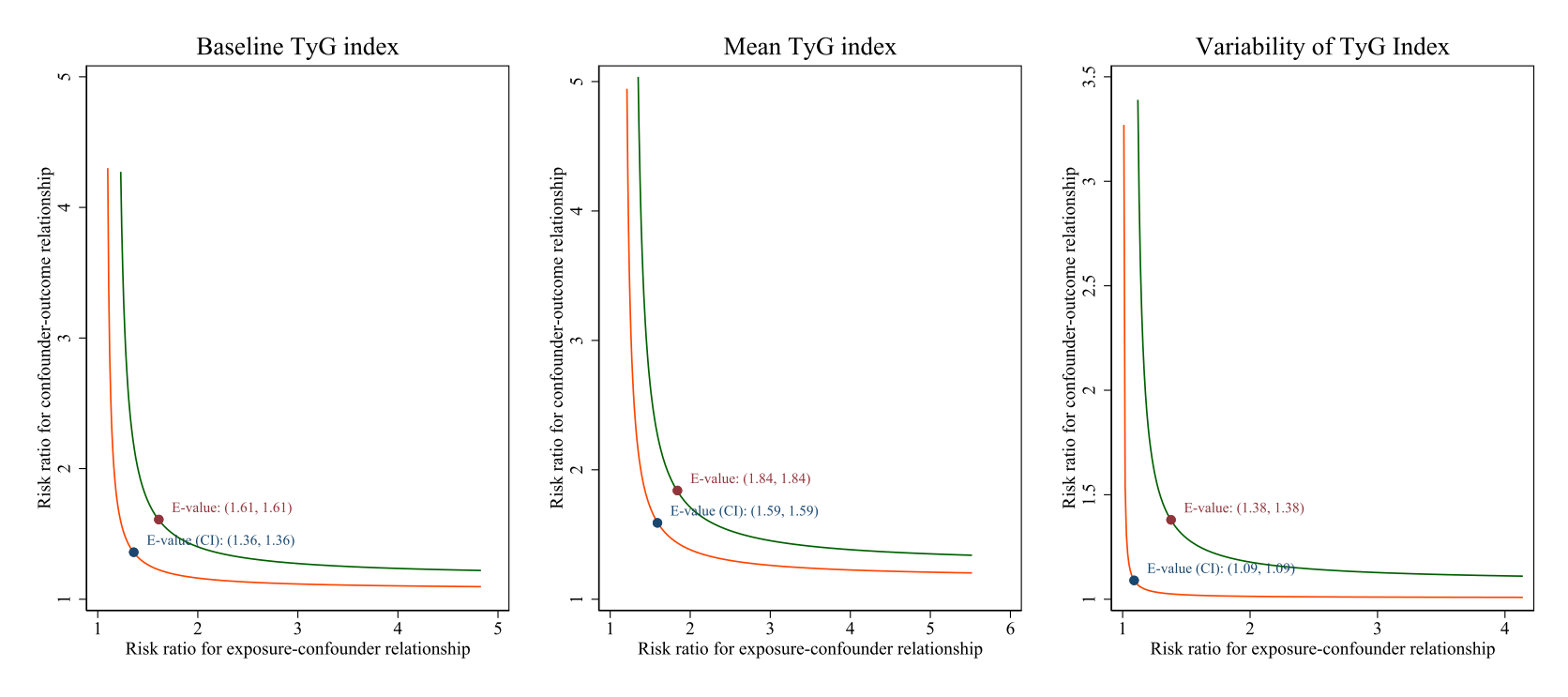


**Figure S7.** E-value for cardiovascular disease according to baseline, mean, and variability of tyg index

| **Table S1. Association between slope of TyG index and the incidence of cardiovascular disease** | | | |
| --- | --- | --- | --- |
|  | **Hazard ratio (95% CI)** | | |
|  | **Decreasing**  **(****< -0.2/year)** | **Stable**  **(-0.2 to 0.2/year)** | **Increasing**  **(> 0.2/year)** |
| No. of cases/Population | 191/3,495 | 1,981/41,258 | 232/4,826 |
| Incidence rate per 1000 person-years | 6.37 | 5.59 | 5.57 |
| Model 1 ^a^ | 1.13 (0.98-1.31) | 1 [Reference] | 1.18 (1.03-1.35) |
| Model 2 ^b^ | 1.12 (0.96-1.31) | 1 [Reference] | 1.04 (0.90-1.20) |
| Model 3 ^c^ | 1.14 (0.98-1.33) | 1 [Reference] | 1.02 (0.88-1.17) |
| ^a^ Adjusted for age and sex. | | | |
| ^b^ Adjusted for age, sex, education, income, current smoking, current drinking, physical activity, body mass index, diabetes, hypertension, chronic kidney disease, and high-sensitivity C-reactive protein. | | | |
| ^c^ Adjusted for covariates in model 2 plus hypercholesterolaemia, low-density lipoprotein cholesterol, and high-density lipoprotein cholesterol. | | | |

| **Table S2. Sensitivity analysis for the association between tertiles of baseline, mean, and variability of TyG index and the incidence of cardiovascular disease** | | | | |
| --- | --- | --- | --- | --- |
|  | **Hazard ratio (95% CI)** | | |  |
|  | **Tertile 1** | **Tertile 2** | **Tertile 3** | ***P* for trend** |
| **Baseline TyG index†** |  |  |  |  |
| Excluding fasting glucose ≥ 7.0 mmol/L or triglycerides ≥ 1.7 mmol/L at baseline | 1 [Reference] | 1.24 (1.10-1.38) | 1.26 (1.11-1.42) | <0.001 |
| Competing risk regression analysis | 1 [Reference] | 1.21 (1.09-1.35) | 1.32 (1.17-1.49) | <0.001 |
| 2-y lag analysis | 1 [Reference] | 1.23 (1.09-1.40) | 1.27 (1.11-1.45) | 0.001 |
| **Mean TyG index†** |  |  |  |  |
| Excluding fasting glucose ≥ 7.0 mmol/L or triglycerides ≥ 1.7 mmol/L at baseline | 1 [Reference] | 1.27 (1.13-1.43) | 1.40 (1.24-1.59) | <0.001 |
| Competing risk regression analysis | 1 [Reference] | 1.25 (1.12-1.40) | 1.40 (1.24-1.58) | <0.001 |
| 2-y lag analysis ^e, f^ | 1 [Reference] | 1.26 (1.11-1.42) | 1.41 (1.24-1.61) | <0.001 |
| **TyG Variability‡** |  |  |  |  |
| Excluding fasting glucose ≥ 7.0 mmol/L or triglycerides ≥ 1.7 mmol/L at baseline | 1 [Reference] | 1.03 (0.93-1.14) | 1.13 (1.02-1.25) | 0.025 |
| Competing risk regression analysis | 1 [Reference] | 1.03 (0.93-1.13) | 1.13 (1.03-1.32) | 0.011 |
| 2-y lag analysis | 1 [Reference] | 1.07 (0.96-1.20) | 1.15 (1.03-1.29) | 0.010 |
| **†**Adjusted for age, sex, education, income, current smoking, current drinking, physical activity, body mass index, diabetes, hypertension, chronic kidney disease, high-sensitivity C-reactive protein, hypercholesterolaemia, low-density lipoprotein cholesterol, and high-density lipoprotein cholesterol. | | | | |
| **‡**Additionally adjusted for baseline TyG index. | | | | |
